# Roles of Voltage-Gated Tetrodotoxin-Sensitive Sodium Channels Na_V_1.3 and Na_V_1.7 in Diabetes and Painful Diabetic Neuropathy

**DOI:** 10.3390/ijms17091479

**Published:** 2016-09-05

**Authors:** Linlin Yang, Quanmin Li, Xinming Liu, Shiguang Liu

**Affiliations:** Department of Endocrinology, The General Hospital of the PLA Rocket Force, Beijing 100088, China; ylynn1028@126.com (L.Y.); xinming2211@126.com (X.L.); tommy19921030@126.com (S.L.)

**Keywords:** diabetes mellitus, painful diabetic neuropathy, Na_V_1.3, Na_V_1.7, dorsal root ganglion neurons, pancreatic islet cells

## Abstract

Diabetes mellitus (DM) is a common chronic medical problem worldwide; one of its complications is painful peripheral neuropathy, which can substantially erode quality of life and increase the cost of management. Despite its clinical importance, the pathogenesis of painful diabetic neuropathy (PDN) is complex and incompletely understood. Voltage-gated sodium channels (VGSCs) link many physiological processes to electrical activity by controlling action potentials in all types of excitable cells. Two isoforms of VGSCs, Na_V_1.3 and Na_V_1.7, which are encoded by the sodium voltage-gated channel alpha subunit 3 and 9 (*Scn3A* and *Scn9A*) genes, respectively, have been identified in both peripheral nociceptive neurons of dorsal root ganglion (DRG) and pancreatic islet cells. Recent advances in our understanding of tetrodotoxin-sensitive (TTX-S) sodium channels Na_V_1.3 and Na_V_1.7 lead to the rational doubt about the cause–effect relation between diabetes and painful neuropathy. In this review, we summarize the roles of Na_V_1.3 and Na_V_1.7 in islet cells and DRG neurons, discuss the link between DM and painful neuropathy, and present a model, which may provide a starting point for further studies aimed at identifying the mechanisms underlying diabetes and painful neuropathy.

## 1. Introduction

Diabetes mellitus (DM) is a chronic disease that affects more than 382 million people worldwide, and this number is expected to rise beyond 592 million by 2035 [1]. In a recent study that aimed to provide the number of deaths attributable to diabetes in the year 2013, it was estimated that 1 in 12 of global all-cause deaths were due to diabetes in adults [2]. With DM becoming increasingly prevalent over time, painful diabetic neuropathy (PDN), as one of its associated major complications, is also rapidly rising.

PDN affects almost 25% of the diabetic population and covers a wide variety of clinical presentations, which involve a significant risk in the quality of patients’ life [3,4,5,6]. The clinical characteristics of PDN range from spontaneous pain to allodynia (pain to a stimulus that is painless under normal conditions) and hyperalgesia (increased pain in response to a painful stimulus) [7,8,9]. The burning, shooting, tingling or lancinating pain of PDN impacts patients’ ability to perform daily activities, disturbs sleep, and causes negative influences on mood, such as anxiety and depression [4,6]. Patients with PDN are consumers of more health care resources, costing up to almost US $17,000 per year in patients with severe pain [10,11]. Even with frequent visits to medical professionals and use of prescription medications, it turns out that the clinical treatment of PDN is often unsatisfactory because the use of high doses of drugs is accompanied by abundant side effects. Therefore, the research of new, safe and effective strategies for the treatment of PDN is necessary. Additionally, the pathogenesis of PDN needs to be elucidated, even though it is complex and difficult. Although several pathogeneses, including metabolic, vascular, autoimmune and oxidative stress-related mechanisms for PDN, have been postulated, the precise cause of neuropathic pain in diabetes remains to be elucidated.

## 2. Main Cells Involved in Diabetes and Painful Diabetic Neuropathy

Diabetes mellitus, commonly referred to as diabetes, is characterized by hyperglycemia due to impaired insulin secretion and aberrant glucagon secretion. Insulin and glucagon are released into the blood by β cells and α cells of the pancreatic islets, respectively, in response to changes in plasma glucose levels [12]. Apparently, pancreatic islet cell dysfunction is critical for the development of hyperglycemia during DM and its associated complications, including PDN. Electrical activity, which is crucial for hormone release by the pancreatic islet cells, is organized by the concerted activity of several different types of ion channels [12,13].

The small diameter Aδ- and C-fibers of dorsal root ganglion (DRG) neurons, also known as pain-sensing sensory neurons (nociceptors), are the target cells of PDN, as they can be sensitized by a variety of mechanisms in response to different pathological conditions associated with diabetes. Increased ectopic discharges of sensory neurons are considered to contribute directly to the development and maintenance of PDN and changes of ion channel activities in DRG neurons play a significant role in peripheral sensitization and nociceptive sensation [14,15].

In previous research, voltage-gated ion channels, such as K^+^ and Ca^2+^ channels, or other factors that may contribute to the pathogenesis of insulin and glucagon secretion, as well as diabetic neuropathic pain, were discussed [12,13,14,15]. In this review, we present the current knowledge on the role of voltage-gated tetrodotoxin-sensitive sodium channels in the perception modulation of DM and PDN.

## 3. Review of Voltage-Gated Sodium Channels

Voltage-gated sodium channels (VGSCs) are integral membrane proteins that allow movement of sodium ions across cellular membranes and are present in many tissue types within human and rodent. VGSCs generate and conduct action potentials and regulate electrical signaling in all types of excitable cells [16,17,18]. The activity of these channels and the movement of charged sodium ions allow them to produce and respond to electrical signals within the excitable cells. Activation, deactivation, and inactivation in VGSCs link various physiological processes to electrical activity by controlling action potentials in different tissues. Because of their distribution throughout the body, VGSCs are implicated in a variety of diseases, including DM and PDN.

VGSCs mediate the influx of sodium ions into the cytosol of cells in response to local membrane depolarization, which results in the generation of the rising phase of an action potential [18]. All voltage-gated channels are formed by a long integral membrane polypeptide, α subunit, and one or more smaller auxiliary β subunits [19]. The ion-selective pore forming the α subunit is the main structure for channel function including voltage-dependent gating and conductance, whereas the kinetics and voltage dependence of channel gating are in part modulated by β subunits [20,21]. In mammals, nine α isoforms (Na_V_1.1–Na_V_1.9, encoded by the sodium voltage-gated channel alpha subunit genes *Scn1A*–*Scn5A* and *Scn8A*–*Scn11A*) and four β subunits (β1–β4, encoded by the sodium voltage-gated channel beta subunit genes *Scn1B*–*Scn4B*) have been identified. The distribution of sodium channel isoforms varies in different tissues and development stages. Based on differential sensitivity to tetrodotoxin (TTX), sodium currents are classified into TTX-sensitive (TTX-S) and TTX-resistant (TTX-R) components. Five members (Na_V_1.1–1.4, 1.6–1.7) of the VGSC family are TTX-S sodium channels, which can be blocked by TTX, while the other sodium channel isoforms are TTX-R sodium channels, which cannot be blocked by TTX [22].

In addition, many differences in sodium channel structure have been shown to arise from the extent of glycosylation. It appears that established mechanisms for structural heterogeneity within a species of ion channel could also contribute to the behavioral heterogeneity among Na^+^ channels. Glycosylation of ion channels may be altered in diabetes. TTX-S VGSCs within DRG neurons (including Na_V_1.3 and Na_V_1.7) can exhibit a spectrum of states of glycosylation [23]. In the previous study of voltage-dependent Na^+^ conductances in small adult rat DRG neurons, conducted by Rizzo et al., they found that, in all small neurons studied, there appeared to be a singular kinetic component of the current, based on sensitivity to the conditioning potential, voltage dependence of activation, and inactivation half-time [23]. The different properties of the slow Na^+^ conductance in different neurons are likely to reflect heterogeneity of the structure of the underlying channel molecule.

## 4. Roles of TTX-S Na_V_1.3 and Na_V_1.7 Channels in Painful Diabetic Neuropathy

The peripheral nociceptive neurons in DRG express a variety of sodium channel isoforms, particularly Na_V_1.3, Na_V_1.7, Na_V_1.8 and Na_V_1.9, each playing a key role in the physiology of nociception. Additionally, their encoding genes have been demonstrated to relate with neuropathic pain [24,25,26]. TTX-S currents induced by both fast and slow voltage ramps increase significantly in diabetic neurons. Na_V_1.3 and Na_V_1.7 are highly TTX-S and their expression levels increased in diabetic animals’ DRG homogenates. Pre-clinical data from non-specific blockers, knockouts and small interfering RNA for these specific subtypes of sodium channels have been revealed to be effective in attenuating hyperalgesia and allodynia.

## 5. Na_V_1.3

During embryonic stage, TTX-S Na_V_1.3 is broadly expressed in DRG neurons of the developing nervous system. Postnatally, the expression of Na_V_1.3 throughout the nervous system decreases dramatically to undetectable levels. However, Na_V_1.3 is reported to be re-expressed within peripheral DRG neurons under certain pathological conditions that involve peripheral nerve injuries such as inflammation and nerve transection [27,28,29,30,31]. A decrease in the expression of TTX-R α subunits and/or an increase in that of TTX-S α subunits (particularly Na_V_1.3) has been previously reported in nerve injury animal models [32,33,34,35]. Painful neuropathy may occur without the symptoms of DM. It is hard to determine to what extent diabetes and VGSC mutations contribute to PDN. Emerging evidence has demonstrated that Na_V_1.3 has a critical role in the development and maintenance of neuropathic pain of PDN. Na_V_1.3 produces sodium currents with rapid repriming kinetics and recovers quickly from inactivation. In addition, owing to the above distinct functional properties, the upregulated expression of Na_V_1.3 channels in diabetic DRG neurons would be expected to increase overall sodium channel density, decrease firing threshold and play an important role in the hyperexcitability of damaged or injured neurons [36,37,38,39]. A previous study has shown that high-level expression of Na_V_1.3 lasted for six months in streptozotocin (STZ)-induced diabetic rats with persistent mechanical allodynia [40]. In addition, long-term hyperglycemia exacerbated inflammatory reactions thus led to the upregulation of Na_V_1.3 [28,41]. 

Hoeijmakers et al. [42] reported that painful neuropathy is not necessarily a complication of diabetes, but may occur before DM, which was observed in two patients. The I739V mutation (c.2215A>G, p.Ile739Val) in Na_V_1.7 has been described in three patients with painful neuropathy, two of whom were found to have diabetes at least a year after the onset of neuropathy. As far as we know, there was no clinical report about painful neuropathy resulting from Na_V_1.3 mutation occurring prior to DM.

Many experts have considered Na_V_1.3 as a suitable target for pain therapeutics. Due to the absence of isoform-selective, effective and safe Na_V_1.3 blockers, research focused on gene therapy. Samad et al. provided evidence for a contribution of Na_V_1.3 to neuropathic pain, and demonstrated the therapeutic potential of Na_V_1.3 knockdown for pain treatment in a rat model. They suggested gene therapy as a potential therapeutic option [43]. In a recent study by Tan et al., they proved the efficacy of Na_V_1.3 knockdown by adeno-associated viral-mediated delivery of small hairpin RNA in an STZ-induced PDN rat model with reduced tactile allodynia, and a concomitant decrease in nociceptive dorsal horn neuron hyperexcitability [44]. Together, these findings demonstrate the functional relevance of Na_V_1.3 misexpression in diabetic neuropathic pain and provide groundwork for developing targeted gene therapy to manage PDN.

Additionally, altered levels of neurotrophin nerve growth factor (NGF) may also take part in the pathophysiology of PDN. NGF is known to regulate the expression of Na_V_1.3. In a previous study, performed by Black et al., they examined the hybridization signal of α-SNS (Na_V_1.8) and α-III (sodium channel III) mRNAs in small DRG neurons from adult rats that had been dissociated and maintained for seven days in the absence or presence of exogenous NGF. They found that NGF participates in the regulation of membrane excitability in small DRG neurons by pathways that include opposing effects on different sodium channel genes including *Scn3A* [45].

## 6. Na_V_1.7

Na_V_1.7, also known as hNE9 or PN1, one of the TTX-S sodium channels, is proved to be an important contributor to pain signaling. Altered expression and gain-of-function mutations of Na_V_1.7 have been described in many studies of neuropathy pain, including PDN. Because of its slow open state and slow closed state inactivation and relatively hyperpolarized activation voltage dependence, Na_V_1.7 amplifies small depolarization below the threshold for the all-or-none action potential [46], thereby setting the gain on action potential electrogenesis and pain signaling by DRG neurons [47]. In PDN, it has been reported that Na_V_1.7 channel expression increased robustly in the DRG neurons of rats and triggered development of hyperalgesia and allodynia [48,49]. The link of Na_V_1.7 and PDN has been supported by the decrease of pain-related behaviors after reduction of Na_V_1.7 in DRG neurons induced via vector-mediated microRNA against Na_V_ α subunits [50], vector-mediated release of γ-aminobutyric acid (GABA) [51], activation of delta opioid receptor [52], or administration of gabapentin [53]. In addition, Na_V_1.7 and Na_V_1.8 are believed to operate in tandem within DRG neurons, with Na_V_1.7 amplifying small stimuli to bring membrane potential to the threshold for activation of Na_V_1.8, which conducts the majority of the inward transmembrane current of an action potential upstroke during repetitive firing [54,55]. In a diabetic model, methylglyoxal depolarized neurons and induced posttranslational modifications of Na_V_1.8, however, it also promoted the slow inactivation of Na_V_1.7 [56]. 

Gain- or loss-of-function mutations in the *Scn9A* gene, which codes for Na_V_1.7, might be the determining pathogenic factor in the development of PDN. To date, at least 19 mutations in the *Scn9A* gene have been reported relating to primary erythromelalgia, which is an exceptionally painful disorder characterized by intermittent severe burning pain, erythema and elevation of temperature in the extremities [57]. Furthermore, gain-of-function mutations in the *Scn9A* gene were found to be linked with paroxysmal extreme pain disorder (PEPD) [58] and idiopathic small fiber neuropathy [59]; whereas, loss-of-function Na_V_1.7 mutations produce congenital insensitivity (or indifference) to pain (CIP), which is a disease in which patients experience painless fractures, lacerations, burns, and tooth extractions, for example [60]. Given that Na_V_1.7 channels are present in both pancreatic β cells and DRG neurons, a new concept, which might explain why some patients have neuropathy before diabetes onset, proposed by Hoeijmakers et al. [42], links the beginning of pancreatic β cell failure and PDN with genetic disruptions on Na_V_1.7 channels. A susceptible genetic background could facilitate generation of Na_V_1.7 mutations, leading to gain-of-function that evokes β cell lesions, and, thereafter, diabetes and hyperexcitability in DRG neurons [42,61]. 

## 7. Roles of TTX-S Na_V_1.3 and Na_V_1.7 Channels in Diabetes

Pancreatic islet cells express TTX-S VGSCs, especially Na_V_1.3 and Na_V_1.7 [62,63,64], which supports the generation of electrical activity. It is demonstrated that Nav1.3 and Nav1.7 channels are expressed within both α and β cells in different amounts, which explains the different properties of Na^+^ currents in both cells [62]. In particular, Zhang et al. found that Na_V_1.3 was the functionally important channel in both types of islet cells, whereas, due to an islet cell-specific factor, Na_V_1.7 channels were locked in an inactive state in mouse islet cells [62].

## 8. Pancreatic α Cells

Because glucagon secretion depends on the generation of Na^+^-dependent action potentials, TTX-S voltage-gated Na^+^ channels play a key role in regulating α cell function [64,65]. In DM, regulation of glucagon release is impaired with its levels inappropriately elevated at high glucose and reduced at low glucose, which might lead to fatal hypoglycemia. Some research indicated that the dysfunction of sodium channels in pancreatic α cells was associated with dysregulation of glucagon secretion in diabetes. In the islet α cells of STZ-induced diabetic mice with hyperglycemia, glucagon content and release was reported to increase due to enhanced Na^+^ current (INa), action potential duration and firing frequency [66]. In contrast, Na^+^ currents were inactivated under hypoglycemic conditions with reduced action potential height which inhibited glucagon secretion [67]. To investigate the underlying mechanism of the antidiabetic effect of VGSC blockers, Dhalla et al. found that glucagon release was mediated by the Na_V_1.3 channels, and selective Na_V_1.3 blockers might provide a novel approach for the treatment of diabetes [68]. Dusaulcy et al. demonstrated that some intrinsic defects were found in α cells and identified the *Scn9A* gene to be involved in glucagon biosynthesis and secretion [69]. The expression of the *Scn9A* gene was decreased in α cells from STZ-induced diabetic mice and insulin treatment normalized Na_V_1.7 [69].

Hoeijmakers et al. [42] hypothesized that Na_V_1.7 mutations chronically depolarize membrane potential, thereby increasing susceptibility to injury of pancreatic β cells and, thus, predisposing the individual to the development of diabetes. According to this hypothesis, diabetes does not necessarily cause peripheral neuropathy, but, on the contrary, both diabetes and neuropathy can occur as a result of Na_V_1.7 mutations, which increase vulnerability to injury in both small nerve fibers and β cells. In fact, Na_V_1.3 mutations possibly play a similar role. Na_V_1.3 channels are present in both pancreatic α and β cells and DRG neurons. The roles of VGSCs in pancreatic cells’ electrical activity are not yet completely understood, but it is likely that VGSCs have important roles in these cells. The chronic membrane depolarization or homeostatic overload and glycosylation influence the pancreatic cells’ activity and the hyperexcitability of DRG neurons. All together, these findings suggest that Na_V_1.3 dysregulation does not necessarily form a direct link between DM and PDN; both diabetes and neuropathy might occur as a result of Na_V_1.3 mutations, which increase vulnerability to injury in small nerve fibers and pancreatic α cells and β cells.

## 9. Pancreatic β Cells

The role of sodium channels in the generation of action potential and effective blockage of the channels using the specific VGSC inhibitor TTX determined TTX-S sodium channels critical for insulin secretion in pancreatic β cells [70,71]. A study showed that insulin secretion by β cells was affected by TTX-S in the mitochondrial membrane, which shaped both global Ca^2+^ and metabolism signals [72]. Furthermore, sodium channels were identified to be potential therapeutic targets in diabetes by analyzing the expression and characteristics of Na^+^ currents in β cells from mice that express green fluorescent protein under the control of the mouse insulin I gene promoter (MIP-GFP mice) [73]. Even though the relative expression of Na_V_1.3 and Na_V_1.7 differs in insulin-secreting β cells, with Na_V_1.7 being the dominant subtype, it has been shown that knocking out the sodium voltage-gated channel alpha subunit 3 *Scn3A* (part of Na_V_1.3 channel) reduces glucose-stimulated insulin secretion in mice [62]. Salunkhe et al. investigated whether modulation of the expression of various VGSC subunits could have an impact on insulin secretion, and found out that VGSCs, especially Na_V_1.3 (encoded by *Scn3A*), are regulated by microRNA-375 in rat insulinoma INS-1 832/13 cells and in primary mouse β cells [74]. Szabat et al. validated the role of the *Scn9A* gene in insulin production by examining Na_V_1.7 knockout mice, and insulin content of islet β cells from these animals had dramatically elevated [75]. Carbamazepine, a sodium channel inhibitor, has been confirmed as a positive modulator, which has protective effects in islet β cells [73,75].

Special cases, as follows, suggest that the presence of VGSC mutations is not only associated with painful neuropathy, but also with DM, where sodium channel genes are involved in insulin or glucagon secretion. *Scn1B* is a major regulatory subunit expressed with Na_V_1.7 protein in mouse pancreatic islets. It was demonstrated that in an *Scn1B* null genetic mouse model, pancreatic glucose-stimulated insulin and glucagon secretion reduced and resulted in severe hypoglycemia [76]. In two particular cases, patients with the Na_V_1.7 I739V mutation (c.2215A>G, p.Ile739Val) were found to have diabetes after the onset of peripheral neuropathy [42,77].

## 10. Conclusions

**Figure 1:**
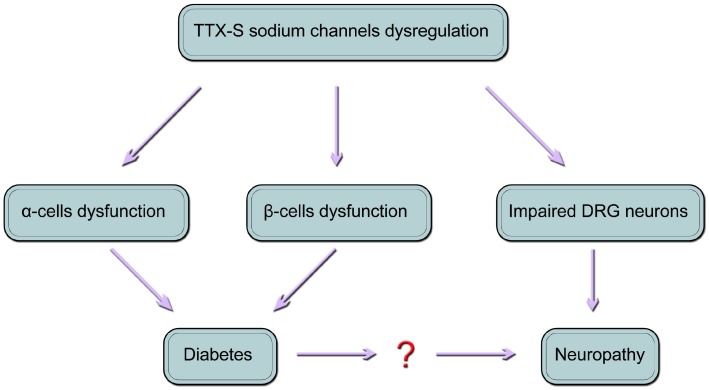
A model of the relationship between diabetes and neuropathy. The dysregulation of tetrodotoxin-sensitive (TTX-S) sodium channels, which are expressed in pancreatic α cells, β cells and dorsal root ganglion (DRG) neurons, leads to diabetes and periphery neuropathy. This model is modified from Hoeijmakers et al. [42].

VGSCs are required for the initiation of action potentials in DRG neurons and pancreatic islet cells, including α cells and β cells. Usually, the roles of TTX-S channels in these three types of cells are separately studied. However, the association between diabetes and neuropathic pain has yet to be analyzed when it comes to the fact that painful form has no relationship to diabetes duration, metabolic control, or the severity of the neuropathy [3]. Based on all the aforementioned studies, it is reasonable to present a model (Figure 1), which is modified from the paper of Hoeijmakers et al. [42]. In this model, we propose that diabetes and peripheral neuropathy are the result of dysregulated TTX-S sodium channels, particularly TTX-S Na_V_1.3 and Na_V_1.7; both diabetes and painful neuropathy might occur as a result of Na_V_1.3 and/or Na_V_1.7 mutations or dysfunction, which increase vulnerability to injury in both small nerve fibers and pancreatic α and β cells.

To what extent the roles of Na_V_1.3 and Na_V_1.7 channels contribute to DM and PDN is unclear, however, it raises doubts as to whether painful neuropathy is caused by diabetes [42,77]. It is necessary to build an adequate stratification of DM patients with neuropathic pain. This new model may provide a starting point for further studies aimed at elucidating the molecular mechanisms of diabetes and painful neuropathy.

There are some limitations in this review. It would be more innovative with evidence based on functional tests. Investigation concerning the functional testing, such as voltage-clamp, patch-clamp and counter-flow of Na_V_1.3 and Na_V_1.7 to support the theories is worthy of future study.
